# Geriatric 8 score as a prognostic factor of the efficacy and safety of oxaliplatin-based chemotherapy in elderly patients with metastatic colorectal cancer

**DOI:** 10.1007/s00384-025-04923-9

**Published:** 2025-05-30

**Authors:** Koki Hara, Wakana Chikaishi, Yunami Yamada, Hironori Fujii, Jesse Yu Tajima, Hirotoshi Iihara, Akitaka Makiyama, Daichi Watanabe, Koichi Ohata, Chiemi Hirose, Ryo Kobayashi, Akio Suzuki, Nobuhisa Matsuhashi

**Affiliations:** 1 Department of Pharmacy, Gifu University Hospital, Gifu, Japan; 2 Department of Surgical Oncology, Gifu University Graduate School of Medicine, Gifu, Japan; 3 Cancer Center, Gifu University Hospital, Gifu, Japan; 4 Laboratory of Advanced Medical Pharmacy, Gifu Pharmaceutical University, Gifu, Japan

**Keywords:** Comprehensive geriatric assessment, Geriatric 8 (G8) score, Metastatic colorectal cancer, Oxaliplatin

## Abstract

**Objective:**

Oxaliplatin (L-OHP) is effective in the treatment of metastatic colorectal cancer (mCRC). However, given concerns about the possible impact of L-OHP-based chemotherapy regimens in the face of physical functional decline, the question of whether they should be actively recommended for elderly patients remains unclear. This study evaluated the relationship between the Geriatric 8 (G8) score, which assesses overall function in the elderly, and the efficacy and safety of L-OHP-based chemotherapy regimens.

**Methods:**

This retrospective study included mCRC patients aged ≥ 70 years who received L-OHP as first-line therapy between January 2017 and December 2022. The primary endpoints were overall survival (OS) and progression-free survival (PFS), and the secondary endpoint was incidence of adverse events (Grade ≥ 2). Patients were classified into high (≥ 14 points) and low (< 14 points) G8 score groups for comparison.

**Results:**

A total of 55 patients were included. Median PFS was significantly longer in the high G8 score group compared to the low G8 score group (12.4 vs. 6.0 months, *P* = 0.034). No significant difference in OS was observed (27.9 vs. 29.8 months, *P* = 0.833). The overall incidence of adverse events was comparable, but nausea incidence tended to be higher in the low G8 score group (0% vs. 25.5%, *P* = 0.096).

**Conclusion:**

The G8 score may serve as a useful prognostic factor in elderly mCRC patients receiving L-OHP. Those with lower G8 scores may be at higher risk of L-OHP-induced nausea.

## Introduction

Colorectal cancer (CRC) primarily affects elderly individuals: the median age at diagnosis is about 66 years [1], 29.4% of patients are diagnosed at age 75 years or older [1]. Systemic therapy for first-line treatment of unresectable colorectal cancer should be selected with consideration to patient age and performance status [2]. In particular, vulnerable patients who are unsuitable for intensive systemic therapy are often considered intolerant to first-line therapy with oxaliplatin (L-OHP) or irinotecan, as well as concomitant therapy with molecular targeted drugs. Elderly patients typically exhibit more age-related organ dysfunction and complications than younger patients, necessitating careful consideration of the risks associated with advanced age in treatment selection [3–6]. In a large population-based cohort of elderly patients with colorectal cancer, for example, those aged 70 years and older experienced higher rates of certain adverse events than patients aged 65–69 years (80.5% vs. 19.5%) [7]. However, clinical trials for metastatic CRC (mCRC) usually involve patients without comorbidities, and few studies focus specifically on elderly patients [8, 9].

L-OHP-based regimens, such as FOLFOX and CapeOX, are established standard chemotherapy regimens for mCRC [2]. A retrospective study by Yamamoto et al. which evaluated the efficacy and safety of regimens containing standard doses of L-OHP as first-line therapy in the advanced elderly (age 75 years and older) with mCRC found that both efficacy and safety were comparable to those in clinical trials not restricted to the elderly [median progression-free survival (PFS), 9.3 months: median overall survival (OS), 38.9 months] [10]. In a post-hoc subgroup analysis of three phase II trials, Fukuchi et al. reported no significant differences in PFS (8.7 months), OS (19.3 months), or frequency of grade 3‒4 toxicities in patients aged 75 years or older receiving L-OHP-based chemotherapy than younger patients [11]. Conversely, while the recently reported open-label phase III trial (JCOG1018) confirmed the superiority of adding L-OHP to fluoropyrimidine (FP) plus bevacizumab (BEV), the authors noted that the proportion of any grade ≥ 3 adverse events was higher in the FP + BEV + L-OHP arm than in the FP + BEV arm (69% vs. 52%), whereas median PFS was 10.0 months and 9.4 months, respectively [12], and concluded that the addition of L-OHP to FP + BEV as first-line treatment did not demonstrate a benefit in older patients. Accordingly, use of age alone as a criterion for determining indications for intensive chemotherapy in CRC patients may not be appropriate.

In current clinical practice, the selection of L-OHP-based chemotherapy regimens for elderly patients with mCRC is typically at the physician’s discretion, with consideration to both age and performance status. Where possible, it is clinically important to identify subsets of elderly patients who can tolerate intensive chemotherapy. Recently, attention has focused on comprehensive geriatric assessment (CGA), which evaluates physical, cognitive, and social factors in the elderly. The American Society of Clinical Oncology (ASCO) recommends using a CGA tool for patients over 65 years initiating chemotherapy [13]. Two commonly utilized tools are the Geriatric 8 (G8) and the Vulnerable Elders Survey-13 [14, 15]. The G8 tool focuses on nutritional management and is considered a sensitive screening tool for elderly cancer patients [16]. A low G8 score has been associated with worse survival in patients with solid tumors [17]. A phase II study evaluating the efficacy of TAS-102 in elderly patients with advanced CRC reported that patients with higher G8 scores (15‒17) had longer PFS and OS than those with lower scores [18]. The G8 score may serve not only as a predictor of chemotherapy efficacy and toxicity — factors which are difficult to assess solely by age and performance status — but also to identify elderly patients who can tolerate intensive chemotherapy. However, current mCRC treatment algorithms are not stratified based on CGA [19].

The aim of this retrospective study was to investigate the relationship between G8 score and the efficacy and safety of L-OHP-based chemotherapy regimens selected as first-line therapy for mCRC in patients aged 70 years or older.

## Patients and methods

### Study design and participants

The study was conducted under a single-center, retrospective design at Gifu University Hospital. Study participants included mCRC patients aged 70 years or over who received L-OHP-based chemotherapy as first-line treatment between January 2017 and December 2022. Patients who did not undergo G8 screening prior to the initiation of chemotherapy were excluded from analysis.

Primary outcomes were OS and PFS, while secondary outcomes included the incidence of adverse events and relative dose intensity (RDI) of anticancer agents. These were compared between the high G8 score (≥ 14) and low G8 score (< 14) groups. Data were extracted from the electronic medical and pharmaceutical records in our hospital’s central database and analyzed retrospectively.

### G8 screening

The G8 tool was developed to screen for elderly cancer patients who might benefit from CGA [14]. It includes eight items, consisting of seven items from the Mini Nutritional Assessment questionnaire such as nutritional status, motor skills, psychological status, medications, and self-rated health, together with a measure of age. The total score ranges from 0 to 17, with a threshold score of 14 indicating good sensitivity and < 14 indicating impairment. In our study, the G8 score was collected by medical professionals from patients before hospitalization to select patients suitable for L-OHP-based chemotherapy.

### Chemotherapy

Patients received treatment with a modified FOLFOX6 (mFOLFOX6) regimen every 2 weeks; a capecitabine (CAP) plus L-OHP (CapeOX) regimen every 3 weeks; or an S-1 plus L-OHP (SOX) regimen every 3 weeks. Bevacizumab (Bmab) and panitumumab (Pmab) were administered as molecular targeting agents. The mFOLFOX6 regimen consisted of a 2-h bolus injection followed by a continuous infusion of L-OHP at 85 mg/m^2^, a 2-h bolus injection of L-leucovorin (*l*-LV) at 200 mg/m^2^, and a 10-min bolus injection of 5-fluorouracil (5-FU) at 400 mg/m^2^, followed by a continuous infusion of 5-FU for 46 h at 2,400 mg/m^2^. The CapeOX regimen involved a 2-h bolus injection of L-OHP at 130 mg/m^2^ and oral administration of CAP at 1,000 mg/m^2^ twice a day from day 1 to day 15, followed by a 7-day rest period. The SOX regimen included a 2-h bolus injection of L-OHP at 130 mg/m^2^ and oral treatment with S-1 at 40 mg/m^2^ twice a day from day 1 to day 15, also followed by a 7-day rest.

### Efficacy of chemotherapy

The primary indicators of the efficacy of L-OHP-based chemotherapy were OS and PFS. OS was defined as the time from the start of treatment to death and PFS as the time from the start of treatment to the first occurrence of disease progression or relapse, or death from any cause.

### Assessment of adverse events

Adverse events associated with L-OHP-based chemotherapy included hematological toxicities such as neutropenia, anemia, and thrombocytopenia, as well as non-hematological toxicities including neuropathy, anorexia, fatigue, nausea, vomiting, diarrhea, constipation, stomatitis/mucositis, and febrile neutropenia. Symptoms of these adverse events were graded according to the Common Terminology Criteria for Adverse Events (CTCAE) version 5.0 [20]. The incidence of adverse events was used as the primary indicator of chemotherapy safety.

### Statistical analysis

Data were analyzed using IBM SPSS version 22 (IBM Japan Ltd., Tokyo, Japan) and R software version 3.5.1 (www.r-project.org). Values of *P* < 0.05 were considered to indicate statistical significance. Patient characteristics were summarized as medians with 25 th and 75 th percentiles for continuous variables and frequencies and percentages for categorical variables. A Kaplan–Meier estimate and log-rank test were used to assess OS and PFS. Hazard ratios between the high and low G8 groups were estimated using a Cox proportional hazards model. Receiver operator characteristic (ROC) curve analysis was performed for each adverse event to determine the optimal G8 score cutoff value by area under the curve (AUC).

### Ethics statement

This study was carried out in accordance with the requirements of the Ethics Committee of Gifu University Graduate School of Medicine and was approved by the Gifu University Graduate School of Medicine Review Committee (Institutional Review Approval Number 2024–056). All procedures performed in studies involving human participants were in accordance with the ethical standards of the institutional and/or national research committee and with the 1964 Helsinki Declaration and its later amendments or comparable ethical standards. We posted information about the trial and how patients could opt out on the hospital’s website.

## Results

### Patient profiles

Eighty patients with mCRC received L-OHP-based chemotherapy as first-line therapy during the study period. Of these, 25 patients were not evaluated for G8 and were excluded from analysis. As shown in Table 1., 55 patients aged ≥ 70 years (33 males and 22 females) were included in this study, with 11 in the high G8 score and 44 in the low G8 score groups. Median age and body mass index (BMI) were 76 years (range: 72‒84) and 21.0 [inter quartile rage (IQR): 18.8‒23.4], respectively.

**Table 1:** Patient characteristics

Sex (male/female), n	33/22
Age (y), median (range)	76 (70–84)
Height (cm)	158.6 (153–165.4)
Body weight (kg)	52.3 (47.8–60.4)
Body mass index	21.0 (18.8–23.4)
G-8 score, n	
High (14–17)	11
Low (0–13.5)	44
Laboratory data	
Neutrophil count (/mL)	3480 (2770–4550)
White blood cells (/mL)	5760 (4840–6745)
Hemoglobin (g/L)	11.9 (10.9–13.5)
Platelets (× 10^9^/L)	23.8 (19.4–27.6)
Albumin (g/dL)	3.9 (3.5–4.2)
Aspartate aminotransferase (U/L)	19 (17–23)
Alanine aminotransferase (U/L)	14 (10–20.5)
Total bilirubin (mg/dL)	0.6 (0.5–0.8)
Serum creatinine (mg/dL)	0.7 (0.59–0.91)
Creatine clearance (ml/min)	56.9 (49.2–70.4)
Chemotherapy, n (%)	
Modified FOLFOX6	9 (16.4)
Modified FOLFOX6 + B-mab	7 (12.7)
Modified FOLFOX6 + P-mab	17 (30.9)
CapeOX	4 (7.3)
CapeOX + B-mab	17 (30.9)
SOX + B-mab	1 (1.8)

Data indicate median values with 25–75 th percentiles unless otherwise indicated. Modified FOLFOX: oxaliplatin with 5-fluorouracil and L-leucovorin, CapeOX: capecitabine plus oxaliplatin, SOX: S-1 plus oxaliplatin, B-mab: bevacizumab, P-mab: panitumumab

Treatment regimens for patients included mFOLFOX6 (16.4%, *n* = 9), mFOLFOX6 + B-mab (12.7%, *n* = 7), mFOLFOX6 + P-mab (30.9%, *n* = 17), CapeOX (7.3%, *n* = 4), CapeOX + B-mab (30.9%, *n* = 17), and SOX + B-mab (1.8%, *n* = 1).

### Comparison of patient profiles between high and low G8 score groups

As shown in Table 2., the high G8 group had significantly higher body weight (60.3 kg vs 51.1 kg, *P* = 0.004), BMI (24.0 vs 20.1, *P* < 0.001), and hemoglobin (13.5 mg/dL vs 11.7 mg/dL, *P* = 0.032) than the low G8 group. No significant differences between the two groups were found for the other items.

**Table 2:** Comparison of patient characteristics between the high and low Geriatric 8 score groups

	G8 ≥ 14 (*n* = 11)	G8 < 14 (*n* = 44)	*P*—value
Sex (male/female), n	7/4	26/18	1.000 ^a^
Age (y), median (range)	74 (70–84)	76 (70–80)	0.492 ^b^
Height (cm)	154.0 (153–165.3)	159.1 (153.3–165.3)	0.535 ^b^
Body weight (kg)	60.3 (55.1–65.1)	51.1 (45.9–59.0)	0.004 ^b^
Body mass index	24.0 (22.9–27.4)	20.1 (18.4–22.3)	< 0.001 ^b^
Laboratory data			
Neutrophil count (/mL)	3480 (3055–3940)	3520 (2740–4613)	0.891 ^b^
White blood cells (/mL)	5540 (4920–6260)	5835 (4570–6888)	0.697 ^b^
Hemoglobin (g/L)	13.5 (12.3–14.0)	11.7 (10.7–13.0)	0.032 ^b^
Platelets (× 10^9^/L)	21.9 (17.4–25.1)	23.9 (19.5–27.9)	0.436 ^b^
Albumin (g/dL)	4.1 (3.9–4.3)	3.85 (3.5–4.2)	0.056 ^b^
Aspartate aminotransferase (U/L)	19 (18.5–23)	19.5 (17–24.3)	1.000 ^b^
Alanine aminotransferase (U/L)	15 (11.5–20.5)	14 (9.8–19.5)	0.569 ^b^
Total bilirubin (mg/dL)	0.7 (0.5–0.8)	0.6 (0.5–0.73)	0.522 ^b^
Serum creatinine (mg/dL)	0.79 (0.68–1.04)	0.70 (0.58–0.89)	0.122 ^b^
Creatine clearance (ml/min)	66.0 (52.0–69.0)	56.8 (48.7–70.6)	0.764 ^b^

Data indicate median values and 25–75 th percentiles. G8: Geriatric 8. Data were statistically compared using the ^a)^ Chi‐square test and ^b)^ Mann–Whitney U test. Significant differences were observed for body weight (*P* = 0.004) and Body mass index (*P* < 0.001)

### Comparison of efficacy of L-OHP-based chemotherapy

**Fig. 1:**
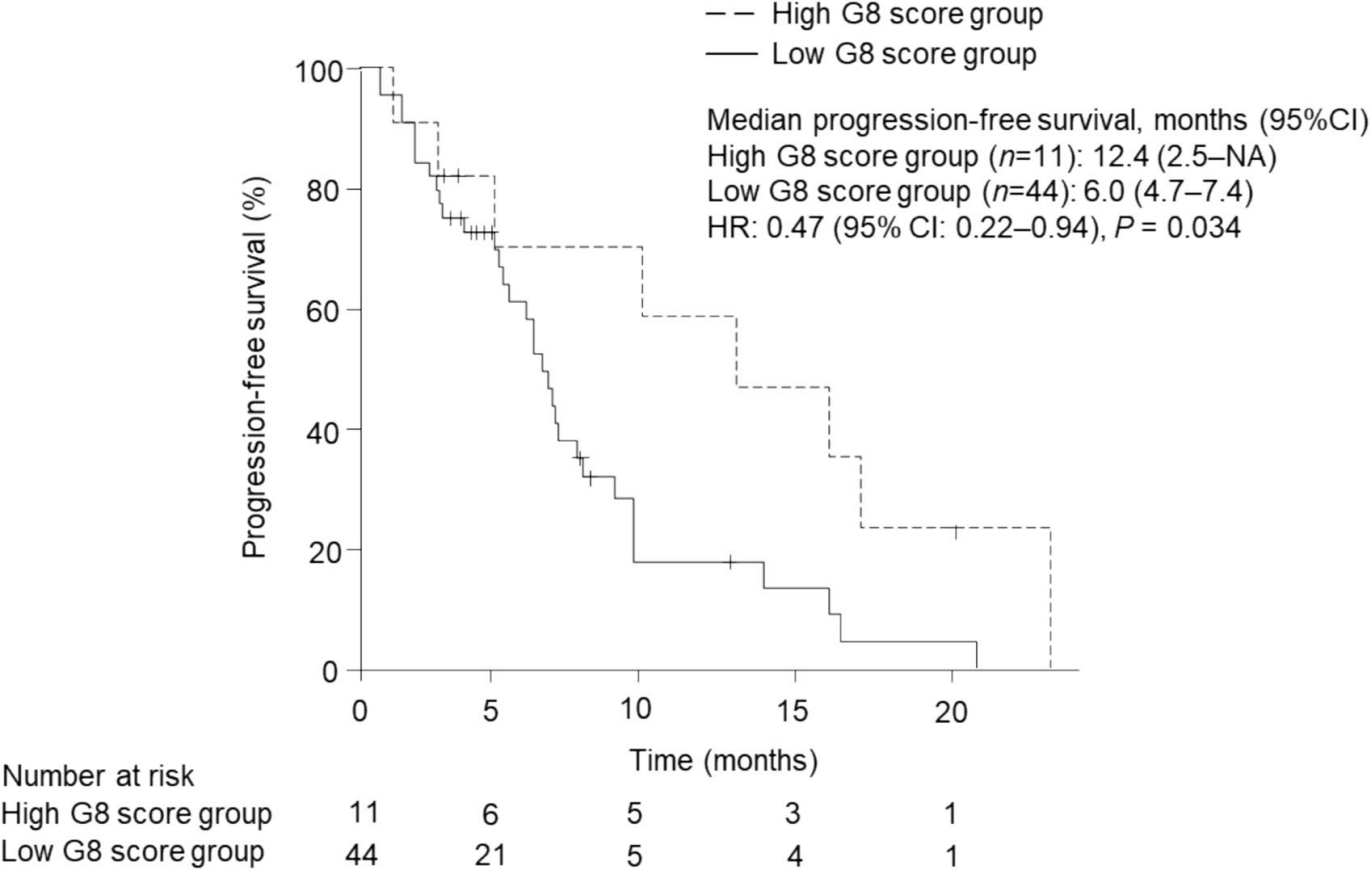
Kaplan–Meier plots comparing progression-free survival (PFS) between the high (*n* = 11, median PFS: 12.4 months, 95% CI: 2.5-NA) and low (*n* = 44, median PFS: 6.0 months, 95% CI: 4.7–7.4) The high G8 score group showed significantly longer PFS compared to the low G8 score group (HR: 0.47, 95% CI: 0.22–0.94, log-rank test *P* = 0.034). CI: confidence interval, HR: hazard ratio

Median PFS was significantly longer in the high versus low G8 score group [12.4 months (95% CI 2.5‒NA) *vs.* 6.0 months (95% CI 4.5‒7.4), *P* = 0.034) [Fig. 1.].

**Fig. 2:**
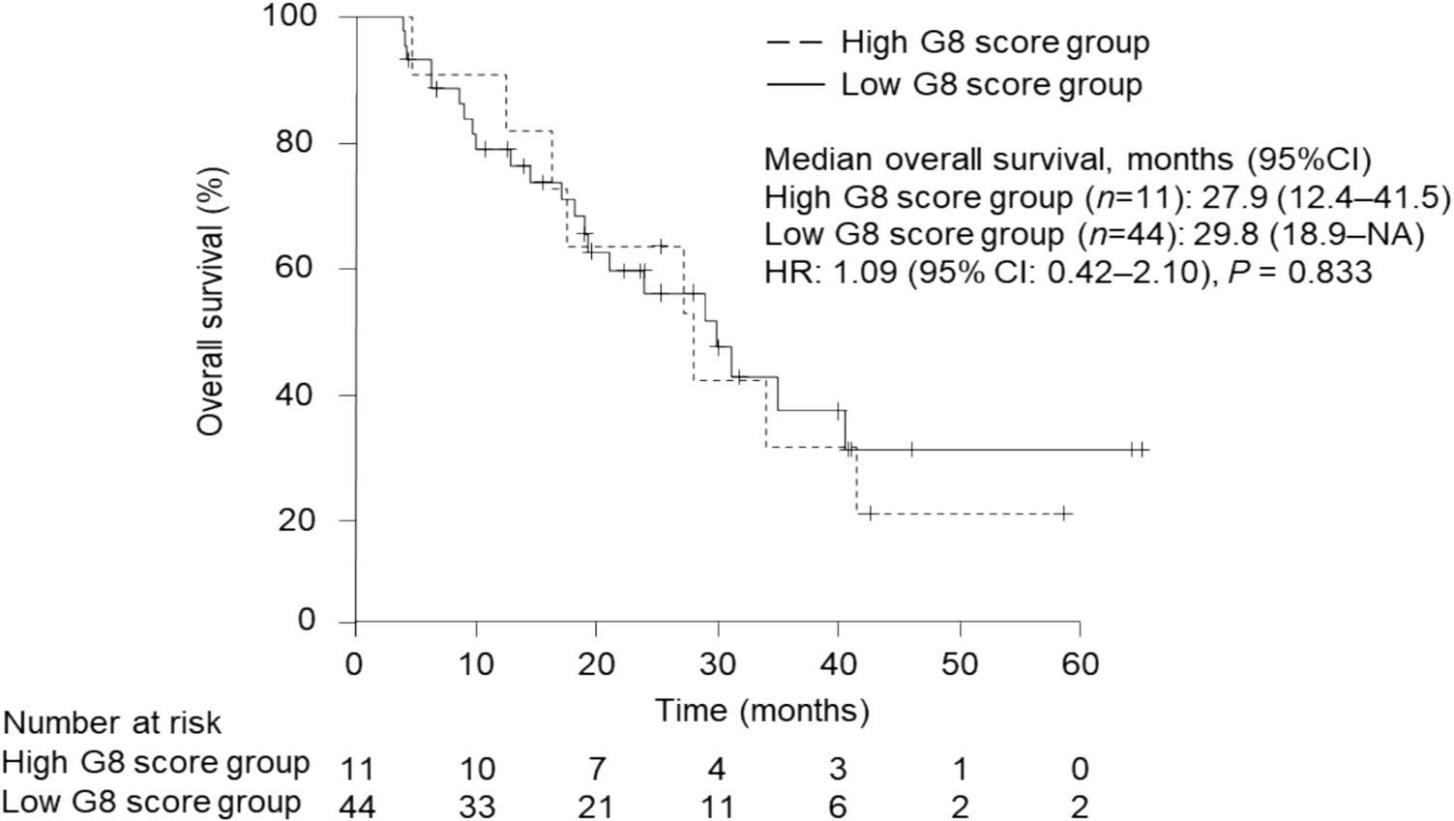
Kaplan–Meier plots comparing overall survival (OS) between the high (*n* = 11, median OS: 27.9 months, 95% CI: 12.4–41.5) and low (*n* = 44, median OS: 29.8 months, 95% CI: 18.9-NA) G8 score groups. No significant difference in OS was observed between the two groups (HR: 1.09, 95% CI: 0.42–2.10, log-rank test *P* = 0.833). CI: confidence interval, HR: hazard ratio

Median OS in the high and low G8 score groups were 27.9 months [95% confidence interval (CI): 12.4‒41.5] and 29.8 months (95%CI: 18.9‒NA), respectively [Fig. 2.].

Median total dose of L-OHP was 517.0 mg (IQR: 333.5‒761.4) (*P* = 0.479) in the high score group versus 487.0 mg (IQR: 391.3‒763.1) in the low G8 score group [Table 3.]. Median RDI of L-OHP was 57.5% (IQR: 26.0‒68.0) in the high G8 score group and 59.8% (IQR: 44.5‒77.1) in the low score group (*P* = 0.118) [Table 3.]. The groups did not significantly differ in either outcome.

**Table 3:** Comparison of oxaliplatin dose between the high and low Geriatric 8 score groups

Oxaliplatin	G8 ≥ 14 (*n* = 11)	G8 < 14 (*n* = 44)	*P*—value
Total dose (mg)	517.0 (333.5–761.4)	487.0 (391.3–763.1)	0.479
Relative dose intensity (%)	57.5 (26.0–68.0)	59.8 (44.5–77.1)	0.118

Data indicate median values with 25–75 th percentiles. Statistical comparison was performed using the Mann–Whitney U test, showing no significant difference in oxaliplatin dose between groups

### Adverse events

As shown in Table 4., adverse events did not significantly differ between the high and low G8 score groups, except for thrombocytopenia. The most common adverse event was neutropenia (55% *vs.* 32%, *P* = 0.293). The incidence of nausea of Grade 2 or higher was significantly higher in the high than the low G8 group (0% *vs.* 25%, *P* = 0.096). The cutoff value of the G8 score for nausea calculated by the ROC curve was 13.5 with an AUC of 0.648 (95% Cl 0.475‒0.816).

**Table 4:** Comparison of adverse events between the high and low Geriatric 8 score groups

	Grade ≥ 2, n (%)		
	G8 ≥ 14 (n = 11)	G8 < 14 (n = 44)	*P*—value
Hematological			
Neutropenia	6 (55)	14 (32)	0.293
Anemia	1 (9)	10 (23)	0.430
Thrombocytopenia	3 (27)	1 (2)	0.002
Non-hematological			
Neuropathy	4 (36)	12 (27)	0.712
Anorexia	2 (18)	13 (30)	0.708
Fatigue	1 (9)	9 (20)	0.667
Nausea	0	11 (25)	0.096
Diarrhea	1 (9)	4 (9)	0.827
Constipation	1 (9)	8 (18)	0.738

Data were statistically compared between the high (*n* = 11) and low (*n* = 44) Geriatric 8 score groups using the Chi‐square test. Significant differences were observed for thrombocytopenia (*P* = 0.002)

## Discussion

We investigated the association between G8 score and the efficacy and safety of L-OHP-based chemotherapy regimens as first-line treatment for mCRC in elderly patients aged 70 years or older. Median PFS was significantly longer in the high G8 score group than in the low group, while median OS did not significantly differ. These findings suggest that G8 score may serve as a prognostic factor for PFS in elderly mCRC patients. In addition, there was a trend toward a higher incidence of nausea in the low G8 group.

Median PFS of the high G8 score group was 12.4 months. In the WJOG4407 and SOFT studies, median PFS for patients with mCRC treated with Bmab + FOLFOX as first-line therapy was 10.7 and 11.7 months, respectively [8, 21]. In the PRIME study, it was 9.6 months with Pmab + FOLFOX [22]. In the GERCOR study, median PFS for CAP + L-OHP and 5-FU/*l*-LV + L-OHP was 8.0 and 8.5 months, respectively [9]. These trials included patients younger than 70 years, yet the median PFS in our high G8 score group was comparable, indicating that selected elderly patients with high G8 scores can benefit similarly from L-OHP-based regimens. Conversely, patients in the low G8 score group had the shorter PFS of 6.0 months.

In the RESPECT study, median PFS for elderly patients receiving regimens without L-OHP was 9.4 months, and 10.0 months with L-OHP-containing regimens [12]. That study concluded that adding L-OHP did not improve outcomes in the elderly. Notably, our low G8 score group had even shorter PFS, suggesting some elderly patients may be less responsive to L-OHP despite receiving a higher RDI.

Based on these findings, the G8 score may be useful in the initial assessment of elderly mCRC patients when selecting first-line therapy. Patients with high G8 scores may benefit from standard L-OHP-based regimens, while those with low scores may be better suited for L-OHP-free or reduced-dose protocols with prophylactic measures against adverse events. Furthermore, patients with low G8 scores may require more intensive supportive care, particularly for nausea. Prospective studies are needed to determine whether G8 scores can effectively guide regimen selection and supportive strategies in this population.

While the SOFT study showed non-inferiority between SOX + Bmab and mFOLFOX + Bmab in first-line treatment of mCRC [21], differences in toxicity profiles among L-OHP-based regimens remain clinically relevant, especially in elderly patients. Future larger studies should analyze outcomes by specific regimen to better understand the interaction between G8 scores and treatment tolerance.

The median total L-OHP dose was 487 mg/m^2^ in the low G8 score group and 517 mg/m^2^ in the high G8 score group (*P* = 0.479), with RDI of 59.8% and 57.5%, respectively (*P* = 0.118). This suggests that frailty, rather than reduced treatment exposure, was the primary driver of poorer outcomes in the low G8 group. Although multivariate analysis adjusting for total dose would be valuable, our sample size limited this approach. This warrants future research with larger cohorts.

It is noteworthy that PFS differed between groups, while OS did not. This may reflect the impact of post-progression therapies. mCRC patients often receive multiple lines of therapy, and OS can exceed 30 months when all cytotoxic agents, including L-OHP and irinotecan, are used [8, 21, 23]. This can be considered one of the factors that makes it difficult to observe differences in OS with first-line treatment alone.

Albumin levels and BMI tended to be lower in the low G8 score group. The G8 score reflects nutritional and physical status [14], and is sensitive in detecting sarcopenia in elderly patients with solid tumors [24]. The Glasgow Prognostic Score, calculated from albumin values, serves as an indicator of nutrition and inflammation status in cancer patients, and is useful for predicting prognosis after chemotherapy for advanced CRC [25]. The low skeletal muscle index, which evaluates sarcopenia at cancer diagnosis, has been associated with worse survival outcomes in patients with solid tumors [26]; thus, nutritional status is closely linked to prognosis in cancer patients. G8 screening may accordingly reflect underlying factors related to nutritional status and physical function that could impact chemotherapy efficacy, such as PFS.

There was no significant difference in the incidence of adverse events between the high and low G8 score groups, except for thrombocytopenia. This finding is consistent with a previous report, and suggests that a G8 cutoff value of 14 may not be clinically useful for predicting OS or serious adverse events in patients with gastrointestinal cancer [27]. Furthermore, in a secondary analysis of randomized controlled trials involving geriatric assessment and management, G8 screening was not identified as a predictor of toxicity, and patients experiencing severe toxicity were more likely to experience functional impairments over time [28].

Thrombocytopenia was significantly more frequent in the high G8 score group. While about 16% of mCRC patients treated with platinum-based regimens develop Grade ≥ 2 thrombocytopenia [29], our observed rate was higher. L-OHP-related thrombocytopenia is typically dose-dependent [30], but RDI was similar between groups. This contradicts the expected pattern of higher hematologic toxicity in frail patients and suggests the need for further investigation. Clinicians should monitor thrombocytopenia even in patients with high G8 scores.

Although peripheral neuropathy incidence did not differ, Grade ≥ 2 nausea occurred in 25% of the low G8 group and none in the high G8 group. While older age is generally associated with lower risk of nausea and vomiting [31], our findings suggest a lower G8 score may increase that risk. ROC analysis identified a cutoff value of 13.5 for nausea prediction, suggesting the conventional cutoff of 14 may not be optimal for this purpose.

This study has several limitations. First, its retrospective, single-center design may have introduced unmeasured confounders. Second, the sample size was small (55 patients over five years), reducing statistical power and generalizability. Third, the BMI criteria used in G8 screening may not be appropriate for elderly Japanese patients, and the definition of polypharmacy (≥ 4) in G8 screening differs from the Japanese definition (≥ 6) [14, 32]. Forth, we did not evaluate interventions based on G8 findings, though such interventions could impact prognosis [33]. Fifth, key prognostic factors such as PS and tumor molecular status (RAS, BRAF, MSI) were unavailable, limiting interpretability.

In conclusion, the G8 score was found to be a prognostic factor in elderly patients with mCRC receiving L-OHP. Additionally, patients with low G8 scores may be at greater risk of L-OHP- induced nausea.

## Data Availability

No datasets were generated or analysed during the current study.
